# Looking to Learn: The Effects of Visual Guidance on Observational Learning of the Golf Swing

**DOI:** 10.1371/journal.pone.0155442

**Published:** 2016-05-25

**Authors:** Giorgia D’Innocenzo, Claudia C. Gonzalez, A. Mark Williams, Daniel T. Bishop

**Affiliations:** 1 Centre for Cognitive Neuroscience, Department of Life Sciences, College of Health and Life Sciences, Brunel University London, Uxbridge, Middlesex, United Kingdom; 2 Department of Life Sciences, College of Health and Life Sciences, Brunel University London, Uxbridge, Middlesex, United Kingdom; University of Exeter, UNITED KINGDOM

## Abstract

Skilled performers exhibit more efficient gaze patterns than less-skilled counterparts do and they look more frequently at task-relevant regions than at superfluous ones. We examine whether we may guide novices’ gaze towards relevant regions during action observation in order to facilitate their learning of a complex motor skill. In a Pre-test-Post-test examination of changes in their execution of the full golf swing, 21 novices viewed one of three videos at intervention: i) a skilled golfer performing 10 swings (Free Viewing, FV); ii) the same video with transient colour cues superimposed to highlight key features of the setup (Visual Guidance; VG); iii) or a *History of Golf* video (Control). Participants in the visual guidance group spent significantly more time looking at cued areas than did the other two groups, a phenomenon that persisted after the cues had been removed. Moreover, the visual guidance group improved their swing execution at Post-test and on a Retention test one week later. Our results suggest that visual guidance to cued areas during observational learning of complex motor skills may accelerate acquisition of the skill.

## Introduction

In the context of motor skill acquisition, demonstrations are one of the most common instructional methods used to convey information to the learner [1]. This process of observing the actions of another person and subsequently adapting one’s own actions accordingly is described as *observational learning* [2]. However, unlike imitation, observational learning is characterized by enduring changes in an individual’s actions [3]. Because of the predominance of observational learning for motor skill acquisition, the processes underlying its efficacy have received considerable research attention. A number of theories and models have been proposed in an attempt to explain the mechanisms that enable individuals to learn novel actions through exposure to an appropriate model. Two models that have been very influential in shaping subsequent research on, and conceptualizations of, observational learning, are, namely Bandura’s Social Cognitive Theory [4,5], and the ecological approach based on Scully and Newell’s direct perception perspective [6]. Although these frameworks have different conceptual underpinnings, both emphasise the importance of attentional processes for effective observational learning.

Social-cognitive approaches to learning [5] emphasize the key roles that cognition and social interactions play in the development and acquisition of novel behaviours. Carroll and Bandura commented that “Virtually all learning phenomena resulting from direct experience can occur vicariously by observing the behavior of others and its consequences” [4]. In his account of social learning theory, Bandura [5,7] noted that, in order for learning to occur via observation, the learner should develop *symbolic representations* of an activity, rather than basic stimulus-responses associations. He proposed four underlying functions that collectively determine the effectiveness of this phenomenon: that the learner should pay attention to relevant information; that retention of the information should occur; that the desired behaviour should be accurately reproduced; and that there should be adequate motivation to do so. Therefore, according to this theory, attentional processes play a pivotal role in learning through observation. Mere exposure to a model guarantees neither appropriate distribution of attention, nor pickup of task-relevant information.

However, Newell [8] observed that social learning theory fails to address the *nature* of the information conveyed by a model. Ecological approaches [8,9] to motor learning advocate that successful learning is contingent upon the observer’s ability to extract relevant information from the environment (e.g., a model) and then to modify his or her behaviour accordingly. Moreover, exogenous guidance of attention towards more task-relevant anatomical regions appears to benefit subsequent execution [10].

The effectiveness of observational learning for motor skill acquisition thus largely depends on the learners’ ability to direct their visual attention to the relevant aspects of the action. This notion presents a potential problem for effective learning in that, when confronted with novel complex motor actions, novices may be unable to identify and focus on the most important elements of the demonstration, thereby failing to pick up important information—which inevitably compromises learning [3]. Consequently, in the present study, we investigated the efficacy of exogenous visual guidance as a means to accelerate novices’ pickup of pertinent visual information and consequently their acquisition of the full golf swing.

### Action Observation and Motor Learning

The role that demonstrations play in learning has been widely explored in the context of sequence learning. For instance, observation of an actor responding to a sequence of stimuli has been found to result in immediate, short-term learning of the observed sequence [11] and the volume of learning accrued through action observation can be comparable to that achieved through physical practice alone [12]. Researchers have also demonstrated the effectiveness of action observation for the learning of complex motor skills including ballet [13], volleyball [14], football [15], cricket bowling [16] and long jumping [17]. Moreover, action observation has proven to be a useful complement to traditional stroke rehabilitation protocols [18].

When designing action observation interventions, it is important to consider a number of factors that may inhibit their effectiveness. These factors include, but are not limited to, the modality of the demonstration (i.e. video, live or virtual model), the viewing perspective, and the characteristics of the task, observer and model [19]. Studies that have examined modality have yielded no differences between live and video models for learning a springboard diving task [20], between live and animated models performing a handstand [21,22], or between live and virtual models demonstrating fly-fishing technique [23]. When viewing the action from a first-person perspective faster skill acquisition may occur, as this perspective requires fewer cognitive resources than does viewing the action from a third-person perspective (i.e. facing the model) or viewing a mirrored image of the action [19]. It has been suggested that third-person and mirrored image perspectives may lead to stronger memory representations and better recall, although studies which have explored these hypotheses have reported equivocal results [24,25]. In contrast, a considerable body of literature shows that the model characteristics affect observational learning of motor skills. Models that possess similar characteristics to the observer—for example, in terms of gender [26] and perceived ability [27]–yield greater performance improvements than models that are dissimilar. However, similarity of skill level seems to be more pertinent than physical characteristics, with skilled models consistently facilitating learning more effectively than unskilled models [28–30].

Although action observation appears to facilitate learning in a variety of contexts, its effectiveness may depend on the observer’s ability to attend to the most informative aspects of the action [31]. It has been shown that, when confronted with complex sport-specific displays, experts attend to more task-relevant regions than novices do. Moreover, novices tend to be preoccupied with elements that are more visually salient than relevant [32]. In observational learning contexts, such ineffective gaze behaviour may prevent or inhibit the acquisition of relevant information. Therefore, by directing the learner’s visual attention to task-relevant regions, observational learning of motor skills may be improved [3]. Accordingly, our aim was to determine whether exogenous guidance may be used to train gaze behaviour during a video modelling intervention, and whether this, in turn, would facilitate novices’ observational learning of the golf swing.

### Gaze Behaviour, Expertise and Skill Acquisition

Researchers have shown that elite performers tend to exhibit more effective gaze patterns than their novice counterparts [33]. Specifically, when trying to anticipate an opponent’s next action, someone who is perceptually skilled often requires fewer fixations of longer duration in order to extract task-relevant information—which indicates an underlying efficiency to their gaze behaviour [34,35]. Moreover, when compared to less-skilled performers, experts are more adept at ignoring redundant/task-irrelevant stimuli [36,37]. Such efficiencies are typically borne out of considerable practice [33,38]. Skill-based differences in gaze behaviours have been demonstrated in contexts other than sport, including air traffic control [39], driving [40,41], medical diagnosis [37] and surgery [42,43]. Accordingly, there is general agreement in the literature that eye movements are an index of learning and skill acquisition to the extent that skilled performers’ gaze is often highly predictive of anticipated future events [44–46].

#### Short-term improvements in gaze efficiency

One theoretical framework that may explain skill-related differences in gaze behaviour and visual search strategies is the Information Reduction Hypothesis [47,48]. According to this theory, individuals learn through practice to select and process only those sources of information that are relevant to the task at hand, and to ignore or inhibit processing of information that is redundant. Haider and Frensch [47] presented participants with a task in which they had to verify the correctness of letters and digits strings. A part of the strings was always redundant to the task, but participants were not informed of this fact. After extensive practice, participants learned to select task-relevant letters while ignoring redundant ones, and the learning effect increased with practice on the task. In a later study, Haider and Frensch [48] recorded participants’ eye movements while they performed the same task and found that redundant letters were fixated progressively less with increased training. Accordingly, they concluded that information reduction occurs at the perceptual, rather than conceptual, level of processing.

Such short-term gains in gaze efficiency have been demonstrated for face perception, even when this process requires individuals to modify their pre-existing gaze behaviour. For instance, gaze strategies for face recognition are learned through extensive experience; initial fixations are typically directed to a region between the eyes and the tip of the nose—an optimal fixation point for the task, as it maximises the pickup of information pertaining to the individual’s identity, gender, and emotional state [49]. However, when Peterson and Eckstein [38] presented participants with a face recognition task in which the mouth was the only feature that discriminated different trials, the optimal gaze strategy was to focus on the mouth region. With practice, the majority of participants gradually shifted their initial fixations to the mouth area, which in turn resulted in improvements in recognition performance and processing efficiency.

### Gaze retraining: accelerating the learning curve

If, as suggested by the ecological and cognitive approaches and the Information Reduction Hypothesis, skill development depends on progressively learning to distinguish and select relevant information, then the question arises as to whether we can accelerate development by directing attention to areas that are task-relevant. This possibility, plus the notion that, with practice on dynamic perceptual tasks, gaze behaviour becomes more predictive and/or selective [50], has led to a groundswell of training programmes aimed at accelerating skill acquisition using exogenous attentional guidance. Cueing attention to task-relevant areas can effectively improve perceptuo-motor performance. For example, Singer and colleagues [51] trained beginner and intermediate tennis players’ anticipatory abilities using either physical practice or verbal tips on how to visually identify and interpret key postural cues. Participants who received verbal cues improved their reaction time and decision accuracy, whereas a physical practice group did not; similar results were reported by Williams and colleagues [52]. Verbal instructions may also improve novice football goalkeepers’ anticipation skill [53].

In sport, verbal cueing paradigms have typically comprised explicit verbal instruction and rules, designed to increase participants’ knowledge and understanding of relevant aspects of the task [51,53]. However, high levels of cognitive processing and explicit knowledge of a skill can hinder performance, leading to skill breakdown [54]. In contrast, implicit methods promote learning without a concurrent accumulation of explicit knowledge, yielding greater automaticity during subsequent skill execution [55,56] and more robustness when the individual must perform under pressure [57,58]. One method by which we may direct learners’ attention to task-relevant regions of a video model without providing explicit information as to why those regions are relevant, is to use exogenous spatial cues. Such cues are highly effective at capturing visual attention in an automatic manner [59].

There is some evidence to suggest that visual cues can effectively aid perception, and consequently learning, of biological motion. Jarodzka, van Gog, Dorr, Scheiter, and Gerjets [60] asked participants to view dynamic and realistic videos of fish swimming. Two experimental groups were provided with visual guidance that was based on an expert marine biologist’s scanpaths. Specifically, for one group the guidance took the form of a red dot, whereas for the other it was presented as a *spotlight* in which the areas fixated were clearly visible and irrelevant areas were blurred out. A control group viewed the videos without guidance. All videos included a spoken description of the locomotion patterns. Subsequently, participants were shown novel videos of different fish swimming according to the previously learned patterns; they were required to identify the different locomotion patterns by identifying the body part used for propulsion (e.g., tail fin) and the way in which this part moves (e.g., undulation). The two experimental groups exhibited more effective visual search patterns and were consequently able to classify the locomotion of novel stimuli more accurately than were control participants. It is conceivable that visual guidance during observational learning of human movement may be similarly effective—but, prior to the present study, this notion was not empirically tested.

One potential issue with exogenous visual guidance is that it has typically been based on the gaze behaviours of experts [60,61]. Since the gaze patterns of experts are more heterogeneous than those of novices, Jarodzka et al. [36] suggested that, when teaching perceptual strategies to novices, it may be preferable to use the perceptual processes of one expert rather than an average of different experts. However, the nature of the information picked up while fixating is less apparent. Therefore, generic rather than specific forms of visual guidance, designed to increase the salience of task-relevant regions, may be more effective in accelerating novices’ information pickup during observational learning.

#### Expanding the attentional ‘zoom-lens’

Increasing the perceptual salience of key information may be beneficial because during observational learning, the various task-relevant features or stimuli are often widely distributed across the visual field. In such cases, consistent with previous research [35], we would expect expert performers, but not novices, to be able to pick up the relevant information probably through their superior ability to extract information through parafoveal and peripheral vision. Therefore, by cueing visual attention to relevant features (i.e., increasing their perceptual salience) we may prompt novices to broaden their attentional focus and thereby distribute attentional resources more effectively. The above suggestion is consistent with the *zoom lens* theory of attention proposed by Eriksen and St. James [62], who showed that the breadth of people’s attentional focus could be manipulated by precueing different locations within a visual display, at varying degrees of eccentricity [63]. The zoom lens model has since been corroborated using fMRI. In a paradigm similar to that used by Eriksen and St. James [62], Muller, Bartelt, Donner, Villringer, and Brandt [64] showed that the extent of activation in participants’ retinotopic visual cortex increased as they expanded their focus of attention—although the *level* of neural activity in any given sub-region of visual cortex tended to decrease, which is consistent with previously observed reductions in processing efficiency as a function of this ‘zooming out’ [62,63].

### The present study

Although researchers have used visual cueing to enhance athletes’ perceptual abilities, the use of such techniques to accelerate observational learning of novel motor skills has largely been neglected. In the present study, we address this issue by investigating the effects of visual guidance on observational learning of the full golf swing. Traditional approaches aimed at developing perceptual skills have involved the direction of attention either via verbal instructions or by the use of an expert model’s scanpaths to guide the observer’s gaze. In contrast, we employed a more generic method of visual guidance, in which experimental participants saw translucent colour patches superimposed over regions of a model golfer’s body and the apparatus (i.e. ball and club), which individually and collectively convey important postural information and spatial relationships for correct setup of the swing. Such implicit methods can arguably be processed in a more automatic and unconscious manner [55], thereby reducing the interference of movement execution that occurs under explicit instruction [65]. Accordingly, since explicit knowledge of the task is not necessary for implicit learning to occur [66], we presented visual guides in the absence of any explicit verbal instruction. We predicted that these cues would attract participants’ overt visual attention, thereby enhancing the pickup of important positional information, without imposing an additional cognitive load for the task. Demonstrations have typically benefited movement form and dynamics rather than movement outcomes [23]; therefore, we assessed improvements in participants’ swing kinematics, rather than on the outcome of their swing (i.e., whether the ball reached a specified target). We hypothesised that, as a result of increased attention to the cued task-relevant information during observation, participants who undertook visually guided observational learning would improve their swing kinematics relative to a group who received no such guidance (free viewing). Finally, implicit learning of complex motor tasks is widely considered to be superior to explicit learning, because explicit knowledge of the rules governing the motor pattern interferes with movement execution by competing for mental resources [65,67]. In contrast, implicitly learned skills are not easily accessible to conscious inspection, are difficult to verbalize and do not place high demands on working memory [68]; hence, they are more resistant to anxiety and pressure, and less likely to be forgotten [67,69]. Since we did not explicitly instruct the visually guided participants to attend to the visual guides, we predicted that these participants would show performance improvements without a concurrent accumulation of additional explicit rules over-and-above those accrued in the free viewing condition [70].

## Method

### Participants

Twenty-one right-handed healthy adults (9 females and 12 males; *M* age = 25.86 yrs; *SEM* = .38 yrs), with normal or corrected-to-normal vision participated. Fifteen participants had no previous experience of performing a golf swing and were randomly allocated to one of three groups: (i) free viewing (FV); (ii) visually guided (VG); or (iii) a control condition. The remaining six participants had played golf once prior to taking part, and were evenly distributed across the three conditions.

**Fig 1:**
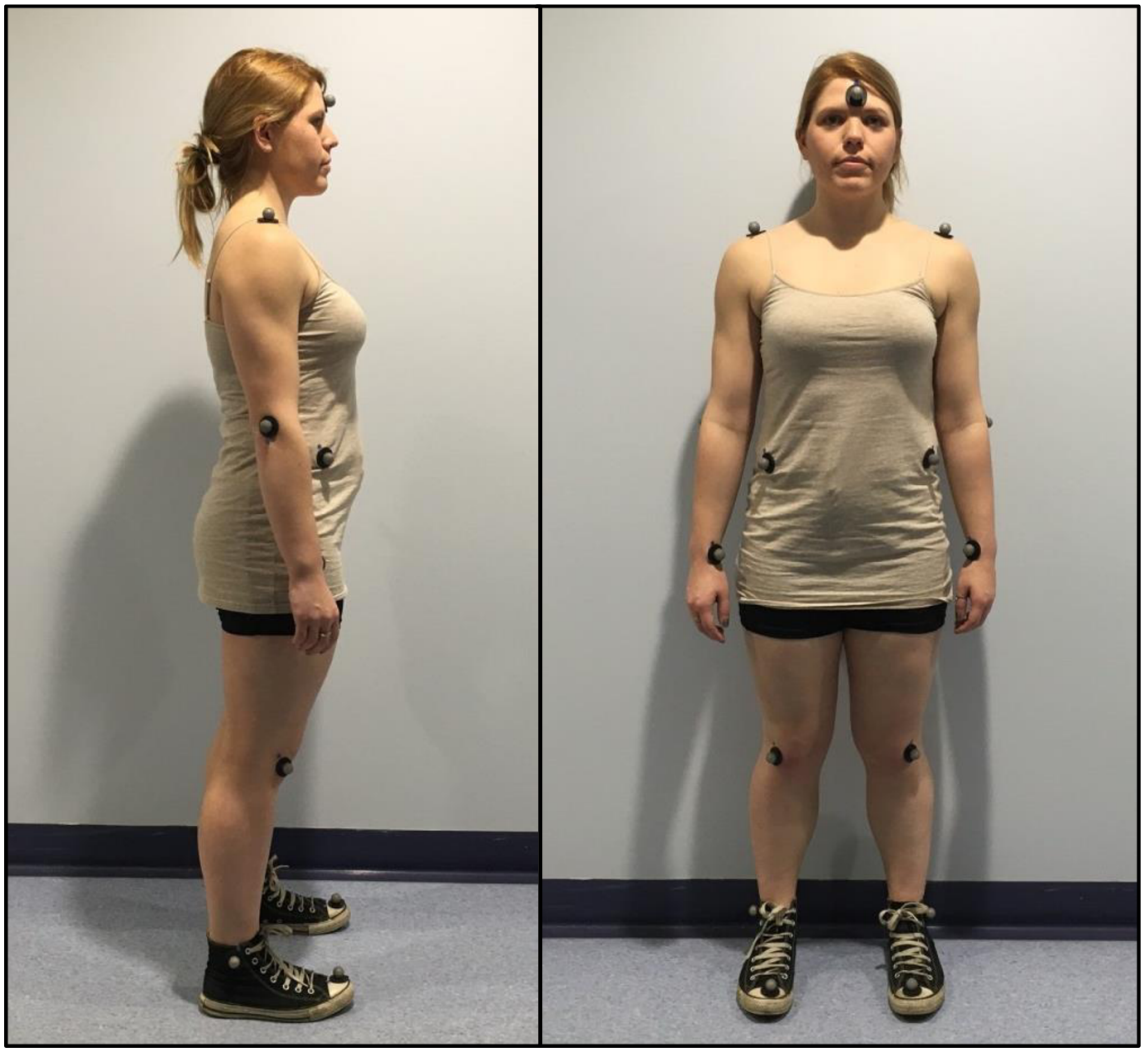
Motion capture reflective marker placement.

**Fig 2:**
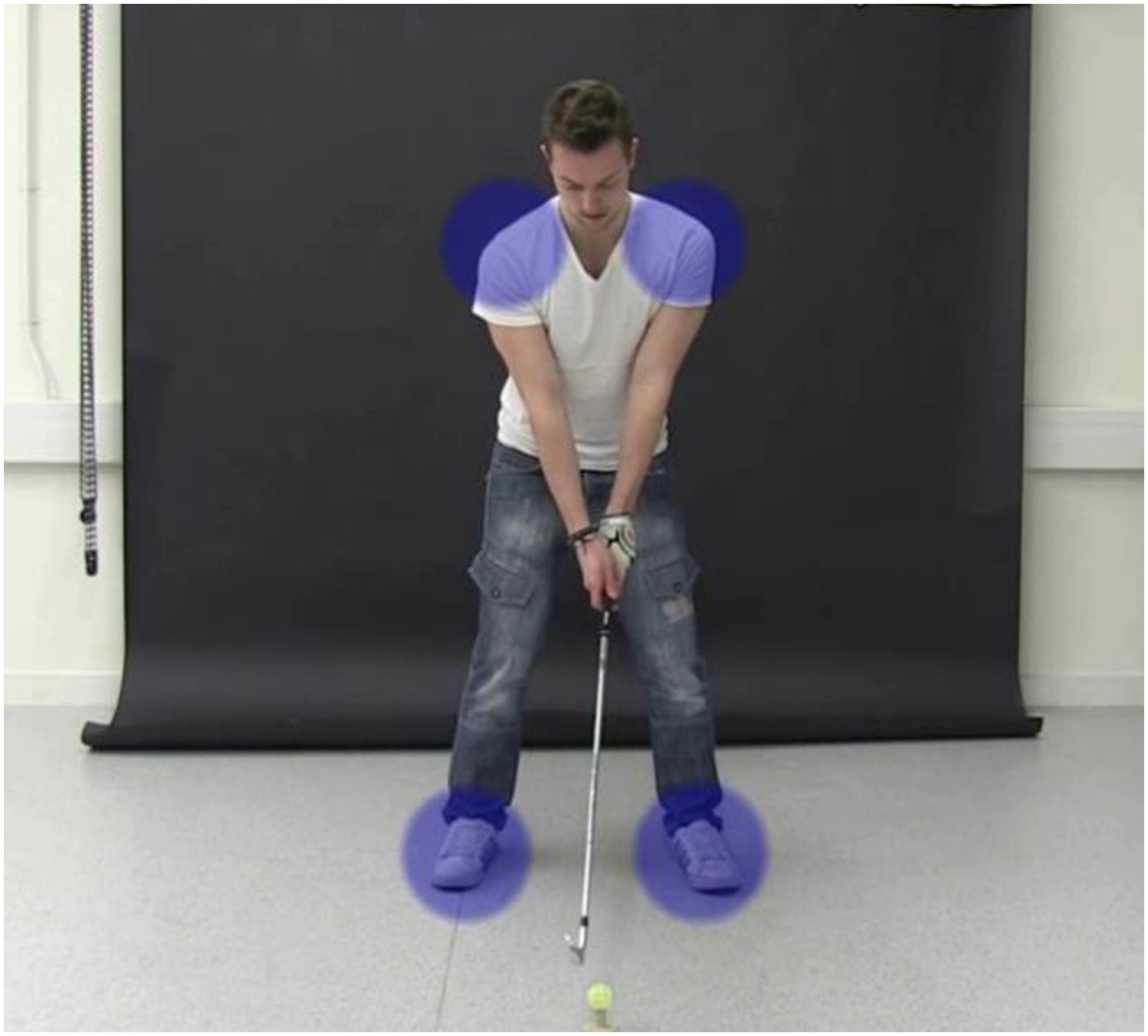
Sample image taken from the intervention video. Colour patches were superimposed on key features during setup.

The Brunel University London Ethics Committee approved the protocol and the consent procedure, and the study was conducted in accordance with the ethical standards of the Declaration of Helsinki. Participants gave their written informed consent prior to taking part. The participant portrayed in Fig 1 and the model portrayed in Fig 2 have given written informed consent (as outlined in PLOS consent form) to publish the pictures.

### Stimuli and Apparatus

Videos of the model (a 25-year-old skilled male golfer with a handicap of 4) were recorded using a Canon HD camcorder, model XF105 (Canon Inc., Tokyo, Japan) and edited using Ulead Video Studio 11 Plus (Ulead Systems Inc., Taipei, Taiwan). Videos displayed a whole-body view of the model from a third-person perspective, with the model facing the participant. Although a first-person perspective may result in faster skill acquisition and better retention [19], such a view would have resulted in considerable loss of pertinent kinematic information.

Participants’ eye movements during observational learning were recorded using a portable eye tracking device (Mobile Eye XG, 30Hz, monocular, ASL, Bedford, Massachusetts). Golf swings were performed using a 6-iron club; motor performance (swing execution) was recorded using a 10-camera, 3-D motion capture software at a 150Hz sampling rate (Cortex v.3.6.1.1315, Motion Analysis, Santa Rosa, California). Fifteen reflective markers were placed on anatomical landmarks important for the correct execution of a golf swing (see Fig 1); one marker was placed on the club head.

### Procedure

The procedure was verbally reiterated to each participant before they completed a demographics questionnaire. Calibration of the motion analysis system was performed before testing each participant. The participant was given two minutes to write down a set of verbal instructions that they would use if they were to explain the correct execution of a golf swing to a novice. This rule formation task was included in order to assess the extent to which participants had developed explicit knowledge of the correct swing execution due to the intervention. The reflective markers were fitted prior to the Pre-test, for which the verbal instructions were as follows: *Please perform 10 full golf swings; your aim is to hit the ball in the direction of the wall while sending it as far as possible*. Because the aim of this study was to determine the effect of a relatively brief intervention, we did not wish to contaminate our data with practice effects. Pilot testing suggested that10 swings would afford some degree of inter-trial consistency of the swing, while keeping physical practice to a minimum. After completing these swings, participants sat in front of a computer screen. The eye tracking device was calibrated using a 9-point grid displayed on the screen. Following successful calibration of the system the participant viewed one of three videos.

The FV group viewed a video of the model performing ten full golf swings; each swing was separated by a 2 s grey screen. Prior to the video the following instructions appeared: *Please watch the following 10 video clips*, *in which a skilled golfer will be executing a golf swing*. *Your aim is to learn about his technique*. VG participants were shown the same video as the FV group, with the exception that they also saw translucent colour patches superimposed on key regions during the setup phase of the swing (e.g., see Fig 2). The instructions prior to the VG video were the same as those for the FV video, with the addition of the following sentence: *Some patches of colour will appear on screen in each clip*. Visual guidance was designed to cue participants’ overt visual attention to the ball and anatomical regions the relative positions of which are fundamental for achieving the correct setup, as consistently emphasised by golf coaches, instruction manuals and websites [71,72]. These included alignment of the head, hands and ball; correct positioning of the ball relative to the feet; an appropriate stance width; and stillness of the head. The control group viewed a video of the history of golf, which contained no reference to the golf swing whatsoever.

After performing ten more full swings (Post-test), participants completed the rule formation task without looking back at their previous answers, in order to assess the extent of explicit rule formation Post-test. Participants’ motor performance was tested again after seven days to assess their retention of the skill. For the Retention test, participants performed ten swings in the absence of any demonstration.

### Data analysis

#### Eye movement data

Eye movement data analyses were conducted using ASL Results Plus (ASL, Bedford, Massachusetts); control participants did not provide eye movement data. Participants’ videos were parsed into swing trials and further divided into three phases, according to the amount of motion involved: (1) a static *setup* phase, in which the golfer ‘addresses’ the ball; (2) a *practice* phase comprising a truncated practice swing, in which the model made minor recalibrations of his positioning; and (3) a *full swing* phase, in which the club head reached speeds of approximately 100 mph. This was done in order to monitor changes in gaze behaviour following the appearance of motion information, as research has shown that motion information automatically attracts visual attention [73]. Moving areas corresponding to the cued areas were then defined as regions of interest for each file in order to derive eye movement data. Dwell times (in ms) were averaged across phases and divided by the total duration of the phase. The resulting dwell time percentages were imported into statistical analysis software (IBM SPSS Statistics v. 20; IBM, Armonk, NY).

#### Kinematic data

Participants’ kinematics were modelled using Cortex (v.3.6.1.1315, Motion Analysis, Santa Rosa, California). A marker set was created in order to label the markers. A linear interpolation function was used to eliminate gaps in the data. Data were smoothed using a 6 Hz low-pass, Butterworth, zero-phase filter. Processed point-light videos were then collated, and each swing was assigned an arbitrary and unique ID. Visual inspection revealed highest inter-trial variability (predictably) at Pre-test, and that this variability was minimal for the final two swings, for the vast majority of participants. Therefore, for the sake of consistency across sessions (i.e. Pre-test, Post-test and Retention) only data from the final two swings of each testing session were compared. Two PGA-qualified professional golf coaches independently rated each participant’s swings at each time point. Specifically, they were asked to provide a numerical rating, on a scale of 1 (very poor) to 10 (excellent), for the participants’ setup and swing execution. For the setup, coaches rated the participants’ posture (i.e., positioning of head, shoulders and knees), plus the relative alignment of these important anatomical landmarks and the feet. For the swing execution, coaches provided a score for the backswing, the club head position relative to the ball at the point of impact, and the follow-through. These scores were summed to obtain a total *performance score* for each participant. The inter-rater reliability of the coaches’ scores was assessed.

#### Rule formation data

The rules formed by the participants were scored by an expert golfer (handicap of 4) and a professional golf coach, who discussed each rule in detail before arriving at a unanimously agreed score. Each rule was assigned a score from 0 to 3 according to the validity and correctness of the information it contained; invalid or incorrect rules were awarded zero points. The experts agreed that the model’s technique represented the benchmark for the highest score, for each of the rules. The validity rating assessed the degree of specificity, correctness and technical detail of the rule. For example, when executing a golf swing, a golf coach will instruct the learner to maintain his or her feet shoulder-width apart. Therefore, inclusion of this rule was assigned the highest possible score of 3. Other rules that referred to the positioning of the feet, however, were only partly correct. For example, “Keep your feet hip-width apart” was assigned a score of 2, as the sentiment is correct, but the anatomical referent is not; “Keep your legs slightly apart” was assigned a score of 1, as it is broadly correct, but without any anatomical referent whatsoever; and “Keep your feet 30 cm apart” was scored as incorrect or invalid (0 points) because it includes misinformation—even to the extent that such instructions could be dangerous (i.e., promoting instability).

For each participant, rule scores were summed in order to derive two separate total scores for, respectively, the number and the validity of rules formed during the Pre- Post- and Retention tests. Swings scores and data from the rule formation task were imported into SPSS for analyses.

## Results

### Eye movement data

**Fig 3:**
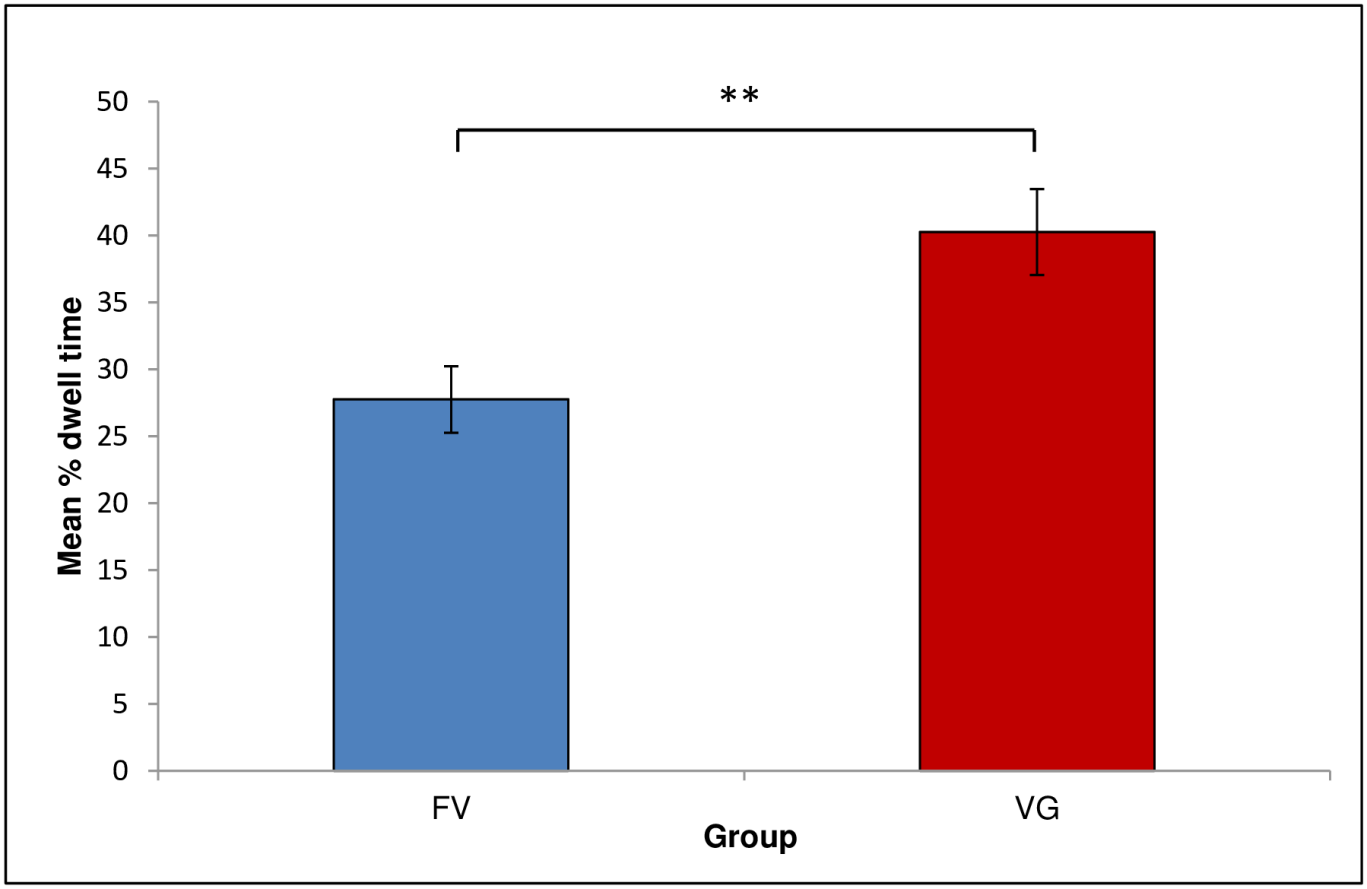
Mean percentage of dwell time on highlighted areas, by Group. Error bars represent standard error of the means. Asterisks indicate a significance level of *p* = .01.

**Fig 4:**
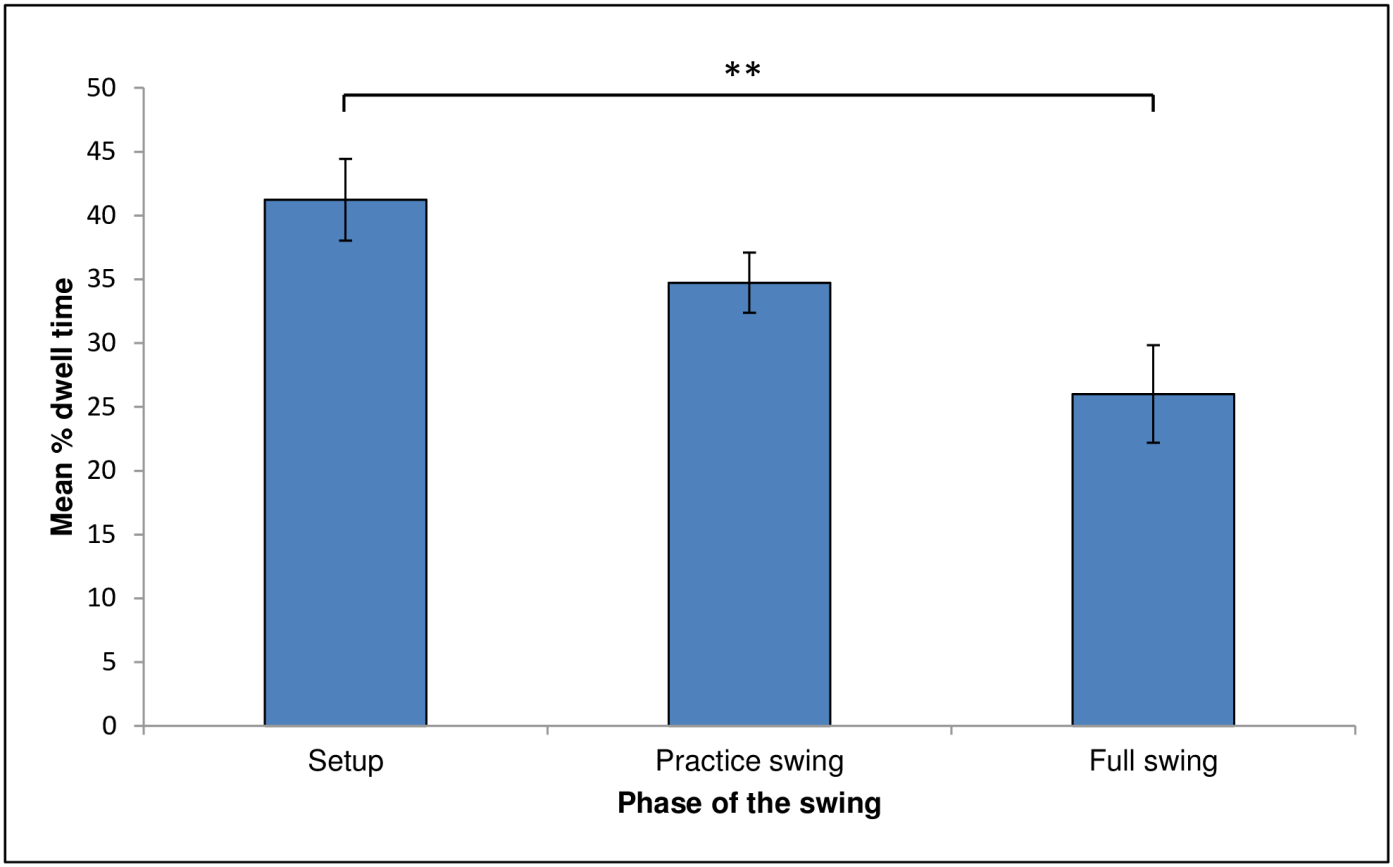
Mean percentage of dwell time on highlighted areas, by Phase. Error bars represent standard error of the means. Asterisks indicate a significance level of *p* = .006.

A Group (FV/VGL) by Phase (Setup/Practice/Full Swing) mixed ANOVA was conducted in order to assess the effects of visual guidance on the total time spent looking at highlighted areas. The interaction between Phase and Group was non-significant. There was a significant main effect of Group, *F*(1,12) = 9.47, *p* = .01, *η*_*p*_^*2*^ = .44. Participants in the VG group (*M* = 40.25, *SEM* = 2.87, 95% CI [33.99, 46.51]) spent more time looking at the areas highlighted by the visual guides than did FV participants (*M* = 27.74, *SEM* = 2.87, 95% CI [21.48, 34]; see Fig 3). A significant main effect of Phase was found, *F*(2,24) = 6.49, *p* = .006, *η*_*p*_^*2*^ = .35. The dwell time on highlighted areas decreased with the progressing phases of the swing, and thus with the increasing amount of motion information contained in the display. Post-hoc tests revealed that, overall, participants spent significantly more time looking at the highlighted areas during the Setup (static) phase of the swing (*M* = 41.23, *SEM* = 3.2, 95% CI [34.26, 48.20]) than during the Full swing (dynamic) phase (*M* = 26.01, *SEM* = 3.82, 95% CI [17.69, 34.34]; see Fig 4).

### Swing execution

A reliability analysis revealed a high consistency between the scores provided by the two coaches, *Cronbach’s α* = .78; discrepancies between the coaches’ scores were found to be very minor and random. These scores were then averaged so as to obtain a single score for each participant’s swing execution in the Pre-, Post-, and Retention tests; analyses were performed on these scores. For the swing scores at Retention, the variances between the three groups were unequal, *F*(2,18) = 3.72, *p* = .045. Moreover, the FV group’s scores for Retention were not normally distributed, *D*(7) = .31, *p* = .041. In order to correct for the absence of normality, a square transformation was applied to all averaged swing scores.

**Fig 5:**
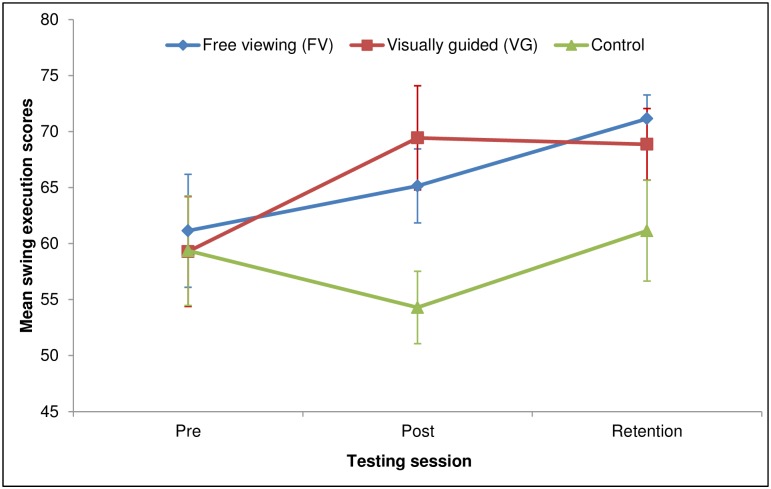
Swing execution scores. Error bars represent standard errors of the means.

A Group (FV/VG/Control) by Time (Pre-test/Post-test/Retention test) mixed ANOVA was conducted to assess the effects of the intervention on motor performance (see Fig 5). There was a significant main effect of Time, *F*(2,36) = 5.70, *p* = .007, *η*_*p*_^*2*^ = .24, and a significant Time x Group interaction, *F*(4,36) = 2.78, *p* = .04, *η*_*p*_^*2*^ = .24. Contrasts revealed between-group differences in the changes from Pre-test to Post-test, *F*(2,18) = 5.80, *p* = .01, *η*_*p*_^*2*^ = .39. Pairwise comparisons with Bonferroni correction were then used to assess the differences between the three groups’ motor performance scores across the Pre-test, Post-test and Retention phases. For the VG group, swing scores were significantly lower during the Pre-test (*M* = 59.29, *SEM* = 4.90, 95% CI [48.9, 69.68]) than in the Post-test (*M* = 69.43, *SEM* = 4.65, 95% CI [61.48, 77.38), *p* = .015 and the Retention test (*M* = 68.85, *SEM* = 3.19, 95% CI [61.69, 76.02]), *p* = .045; scores achieved during the Post-test did not differ from those at Retention. For the FV group, performance scores during the Pre-test (*M* = 61.14, *SEM* = 5.04, 95% CI [50.75, 71.53]) did not differ from those achieved during the Post-test (*M* = 65.14, *SEM* = 3.31, 95% CI [57.19, 73.10]). However, there was a significant difference between the scores in the Pre-test and those in the Retention test (*M* = 71.14, *SEM* = 2.12, 95% CI [63.98, 78.31]), *p* = .034. No significant differences were found between the Control group’s Pre-test (*M* = 59.36, *SEM* = 4.89, 95% CI [48.97, 69.75]), Post-test (*M* = 54.29, *SEM* = 3.23, 95% CI [46.33, 62.24]) and Retention test (*M* = 61.14, *SEM* = 4.49, 95% CI [53.98, 68.31]) scores. Only VG participants significantly improved their motor performance from Pre- to Post-Post-test. Moreover, this improvement persisted at Retention (i.e., 7 days after the first testing session), suggesting that the effects of the visual guides were relatively enduring.

### Rule formation task

**Fig 6:**
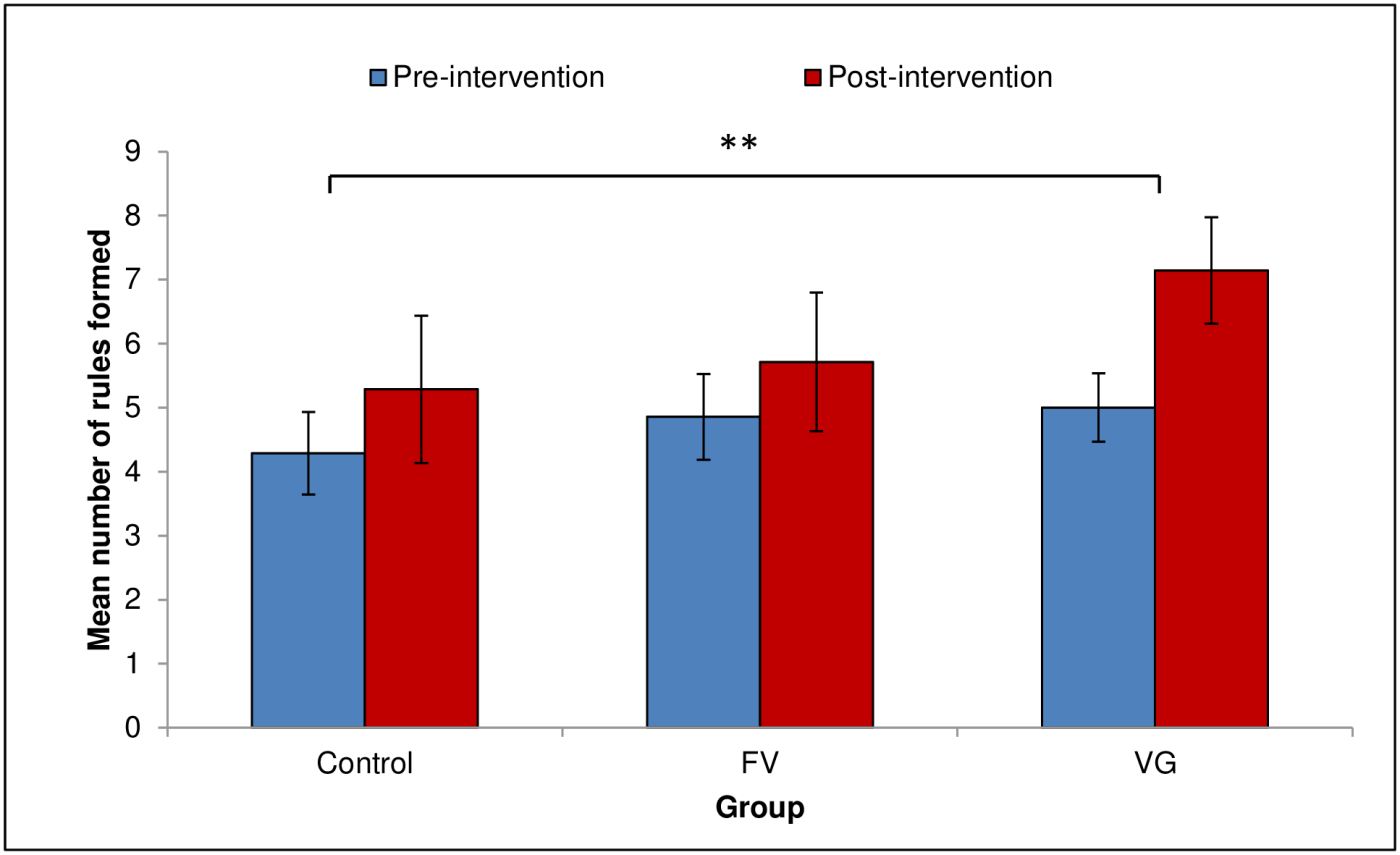
Rules formed. Mean number of rules formed by the three groups before and after the intervention. Error bars represent standard errors of the means. The asterisk indicates significant differences, *p* = .002.

Two separate mixed ANOVAs were conducted in order to explore differences between the three groups’ performances on the rule formation task before and after the intervention. There were no significant main effects or interactions for the quality of rules formed. With regard to the number of rules formed, a Group (FV/VG/Control) x Time (Pre- and Post-test) mixed ANOVA revealed a significant main effect of Time, *F*(1,18) = 12.51, *p* = .002, *η*_*p*_^*2*^ = .41; participants formulated more rules after (*M* = 6.05, *SEM* = .6, 95% CI [4.80, 7.30]) than before (*M* = 4.71, *SEM* = .36, 95% CI [3.96, 5.47]) the intervention (see Fig 6). Some examples of the rules formed by the participants are provided in Table 1.

**Table 1:** Examples of rules formed.

Rule formed	Validity
Bend your back slightly	3
Twist your foot when hitting the ball	0
Weight equally distributed on both feet	3
Rotate your hips through the motion	3
Keep your legs slightly apart	1
Follow through with the swing	3
Right arm should bend, left arm should always be straight	0
Align your feet	2
During the movement, the right leg should follow the direction of the club	0
Line up the club with the ball before the shot	3
Slightly lift your left foot to accompany the swing movement	0
Keep your eyes on the ball throughout	3
Look to the side as you hit the ball	0
When swinging, keep front leg still	0
Keep your feet in line with your shoulders	3
Focus on technique rather than power	3
Keep your head down until the ball has left the tee	3
Keep your head in line with the ball	3
Keep your head straight ahead and don’t look at the ball when it’s being hit	0
Watch your club as it follows through to where you’re hitting the ball.	0
Follow through when swinging the club	3
Keep your eyes on the ball	3
Look forwards as you hit the ball	0
When you swing the club, keep the lower arm straight	0
Bend the arm at the top of the club	1
Keep your feet slightly apart from each other	0
Keep your feet apart	1
Bend your knees slightly	3
Keep your legs straight	0
Pull the club back behind your left arm	1
Swing comes up past shoulder and continues round to opposite shoulder	2
Foot twists as you finish the strike	2
After striking the ball, turn your back foot to point in the direction of the ball	3
Feet twist after contact	0
Swing the club transferring your weight from back to front foot	2

## Discussion

Our data show that, by guiding novices’ attention to task-relevant aspects of a video model’s performance, we accelerated their observational learning of a motor skill—in this case, the full golf swing. Consistent with previous findings [31,60,74,75] and our predictions, exogenous cues were successful in directing participants’ overt visual attention to task-relevant regions of the modelled action in the absence of any explicit instructions to do so. Both experimental groups improved their performance significantly from Pre-test to Retention, whereas the control group evinced no such improvement. Thus, action observation per se promoted learning of the golf swing irrespective of the visual cues. However, only participants who received visual guidance improved their execution of the swing immediately post-intervention.

The above finding is consistent with social cognitive and ecological accounts of motor learning. Both these approaches emphasise the key role that attentional processes play during observational learning: unless the learner attends to and extracts the relevant information, mere exposure to a model does not guarantee learning [5,8]. Accordingly, when learning from complex visual displays, novices often fail to pick up task-relevant information because they focus their attention on features that are perceptually salient, regardless of their relevance to the task at hand [31]. As argued by Bandura, “People’s perceptual sets, deriving from past experience and situational requirements, affect what features they extract from observations and how they interpret what they see and hear” [7]. Our participants had negligible to no golfing experience, and so their attention was likely to be attracted by visually salient or socially relevant areas, such as the fast-moving club or the model’s face, respectively. In order to avoid this, we superimposed translucent colour cues on key areas of the model’s body and on the ball, the relative positions of which are typically highlighted by golf coaches and instructional manuals as fundamental to a correct setup [71,72]. These guides were successful in directing golf novices’ attention to these low-salience but highly relevant features, which resulted in participants spending more time looking at these individually and/or collectively informative regions, reflecting the strategies typically adopted by experts [33,36,37].

Unlike the phenomenon of inattentional blindness, whereby fixations do not guarantee information pickup [76], there was a high degree of correlation between the extent to which key regions were fixated and motor performance. The VG participants’ kinematics improved, suggesting that these participants *were* able to pick up relevant information pertaining to correct positioning and mechanics. Therefore, as argued by social cognitive and ecological motor learning theories, attentional processes do seem to play a central role in determining the extent of information pickup, and thus the effectiveness of action observation for skill acquisition. The performance improvements observed in the visually guided group immediately after the intervention suggest that the visual guides helped participants select and focus on the relevant information. As suggested by the Information Reduction Hypothesis [47], expertise is reflected in an ability to select task-relevant information; hence, the technique employed here may act as a *shortcut* for developing expert-like gaze behaviours.

The free viewing group’s improvements in performance are also noteworthy. Despite the lack of significant improvements immediately following the intervention, free viewing participants’ performance scores at Retention were even higher than those of the visually guided participants, albeit not significantly so. This result is consistent with the notion that a third-person perspective, whereby the learner faces the model, can promote long-term learning and retention of motor skills [19]. Thus, irrespective of the presence of visual guides, observational learning led to superior performance during a delayed Retention test, relative to controls. It is also possible that the very low number of demonstrations provided to participants was simply insufficient for the post-intervention effect on the visually guided participants’ performance to manifest itself at Retention, relative to that of the free viewing group. In fact, both the frequency of demonstrations and the learner’s control over this frequency have been proposed to be important for effective observational learning [19,77]. However, what should be emphasised is that the intervention employed here, despite being ephemeral in nature, was nevertheless effective in *accelerating* learning of the modelled skill. Therefore, such interventions may represent a way to promote efficiency of learning by reducing the number of observations needed to acquire novel motor skills. Another factor that may have affected the present results can be found in the model’s characteristics: our sample comprised both male and female university students, whereas the video model was male—albeit age-matched to the participants. A limitation of the present study is that we did not ask participants to what extent they perceived the model to be similar to themselves. Since perceived similarity has been reported to facilitate observational learning [26,27], this factor should be taken into account in future studies. Specifically, researchers investigating observational learning effects should measure the degree of perceived similarity so as to determine whether this characteristic systematically affects learning (e.g., as a covariate).

Gaze data analyses showed that the time spent fixating on the highlighted areas decreased as the swing progressed—as the model’s overall movement increased. This finding is not surprising for two reasons: first, researchers have shown that, during dynamic scene viewing, motion strongly attracts gaze [73]; and second, the visual cues shown to the VG group were only present during the first few seconds of the setup phase and therefore disappeared prior to swing execution. Although both experimental groups attended to the highlighted regions to some degree, the VG group spent significantly more time looking at these areas even after the cues had disappeared. However, in the absence of any visual guides, and as the golfer started to move, it is likely that rapidly moving elements (e.g., the hands) automatically captured attention.

The rule formation task showed that, overall, participants tended to formulate more rules Post-test. This increase happened in the absence of any explicit instructions and was more pronounced in the VG group, suggesting that rule formation may actually have been *enhanced* by the presence of the cues. A viable explanation for this finding is that the visually guided participants, despite being unaware of the informativeness of the visual guides, may nevertheless have perceived them to be important, and consequently tried to make sense of them. However, a similar trend was observed in the control participants, despite the fact that their video contained no reference to the golf swing, and so may simply reflect the fact that, Post-test, participants were able to create rules because they had performed the swing several times. This result, coupled with the finding that the increase in the number of explicit rules formed yielded no corresponding increase in their validity, suggests that the increase in the number of rules did not result from the participants’ increased knowledge of the golf swing. In fact, as pointed out by Abernethy and colleagues [70], one drawback of using questionnaires to assess explicit rule formation is that the number of rules formed is heavily contingent upon the nature of the instructions provided to the participant.

Although participants did try to interpret what they had seen in the videos by assigning specific rules to different anatomical areas, it is possible that they were unable to correctly process and interpret the visual information that they received. The results of the motor performance, however, suggest that since both the VG and the FV groups performed better in the Retention session, participants in these groups were able to extract meaningful information from the videos of the model via implicit learning mechanisms. This type of implicit learning may be preferable to explicit learning. As opposed to skills that have been learned implicitly, explicitly learned skills are disrupted under conditions involving anxiety and pressure, and do not lend themselves well to transfer tests [78]. In the case of golf, a suitable transfer test would be the use of a different club; notably one that engenders different kinematics from that used in the acquisition phase.

In conclusion, we were able to demonstrate that a brief intervention comprising exogenous orienting of overt visual attention to task-relevant regions of a video model successfully accelerated initial acquisition of the full golf swing. Our finding has important implications for the development of observational training programmes aimed at teaching novel motor skills to novices. Although traditional sport training programmes mainly focus on long-term retention and transfer of the skill [79], the usefulness of cueing techniques should not be underestimated. Our results show that the application of simple visual guides during action observation of a complex motor skill was effective in guiding novices’ attention to key areas, which in turn accelerated initial acquisition of the observed skill. By integrating such cueing methods into traditional training programmes, we may improve the effectiveness of instructional techniques by aiding learners to identify and focus on important sources of information, thus achieving the correct movement form in less time than would be required in the absence of such attentional guidance. Action observation-based training programmes comprising a visual guidance component may represent a time- and cost- effective method of maximising the efficacy of observational practice, by optimising the learner’s attentional processes and resultant information extraction.

Previous examinations of cueing techniques—most notably for improving anticipation skill in sport—have reported contrasting results, to the extent that a consensus on which is the most effective method has not yet been reached [70]. Thus, future research focusing on the acquisition of motor skills through observational learning should directly compare cue types to ascertain their effectiveness for learning. Moreover, research is needed to determine whether simple visual guidance interventions such as the one employed in the present study may enhance observational learning in non-sporting contexts. The beneficial effects observed herein suggest that this intervention may be relevant not only for teaching and learning of sport-specific skills, but also for motor rehabilitation programmes (e.g., in stroke recovery). There is already evidence that action observation can improve motor function in patients suffering from motor deficits following stroke, and that these improvements are greater than those observed after traditional rehabilitation treatments that only employ physical practice [18]. Therefore, the introduction of a visual guidance element may accelerate patients’ reacquisition of previously-learnt skills. The simplicity and brevity of the intervention used herein suggest that it may be applied effectively in a wide variety of contexts—sporting and otherwise.

## Supporting Information

S1 File Full dataset for the performance scores and rule formation task. (SAV)

S2 File Dwell time data. (SAV)
